# Transcriptomic Profiling of Tumor-Infiltrating CD4^+^TIM-3^+^ T Cells Reveals Their Suppressive, Exhausted, and Metastatic Characteristics in Colorectal Cancer Patients

**DOI:** 10.3390/vaccines8010071

**Published:** 2020-02-06

**Authors:** Varun Sasidharan Nair, Salman M Toor, Rowaida Z Taha, Ayman A Ahmed, Mohamed A Kurer, Khaled Murshed, Madiha E Soofi, Khalid Ouararhni, Nehad M. Alajez, Mohamed Abu Nada, Eyad Elkord

**Affiliations:** 1 Cancer Research Center, Qatar Biomedical Research Institute (QBRI), Hamad Bin Khalifa University (HBKU), Qatar Foundation (QF), P.O. Box 34110, Doha, Qatar; vsnair@hbku.edu.qa (V.S.N.); mstoor@hbku.edu.qa (S.M.T.); rotaha@hbku.edu.qa (R.Z.T.); kouararhni@hbku.edu.qa (K.O.); nalajez@hbku.edu.qa (N.M.A.); 2 Department of Surgery, Hamad Medical Corporation, P.O. Box 34110, Doha, Qatar; aahmed40@hamad.qa (A.A.A.); mkurer@hamad.qa (M.A.K.); mabunada@hamad.qa (M.A.N.); 3 Department of Pathology, Hamad Medical Corporation, P.O. Box 34110, Doha, Qatar; kmurshed@hamad.qa (K.M.); madiha.soofi@gmail.com (M.E.S.); 4 Biomedical Research Center, School of Science, Engineering and Environment, University of Salford, Manchester M5 4WT, UK

**Keywords:** colorectal cancer, tumor microenvironment, T cell immunoglobulin mucin-3, exhausted T cells, metastasis

## Abstract

T cell immunoglobulin mucin-3 (TIM-3) is an immune checkpoint identified as one of the key players in regulating T-cell responses. Studies have shown that TIM-3 is upregulated in the tumor microenvironment (TME). However, the precise role of TIM-3 in colorectal cancer (CRC) TME is yet to be elucidated. We performed phenotypic and molecular characterization of TIM-3^+^ T cells in the TME and circulation of CRC patients by analyzing tumor tissues (TT, TILs), normal tissues (NT, NILs), and peripheral blood mononuclear cells (PBMC). TIM-3 was upregulated on both CD4^+^ and CD3^+^CD4^−^ (CD8^+^) TILs. CD4^+^TIM-3^+^ TILs expressed higher levels of T regulatory cell (Tregs)-signature genes, including FoxP3 and Helios, compared with their TIM-3^−^ counterparts. Transcriptomic and ingenuity pathway analyses showed that TIM-3 potentially activates inflammatory and tumor metastatic pathways. Moreover, NF-κB-mediated transcription factors were upregulated in CD4^+^TIM-3^+^ TILs, which could favor proliferation/invasion and induce inflammatory and T-cell exhaustion pathways. In addition, we found that CD4^+^TIM-3^+^ TILs potentially support tumor invasion and metastasis, compared with conventional CD4^+^CD25^+^ Tregs in the CRC TME. However, functional studies are warranted to support these findings. In conclusion, this study discloses some of the functional pathways of TIM-3^+^ TILs, which could improve their targeting in more specific therapeutic approaches in CRC patients.

## 1. Introduction

Colorectal cancer (CRC) is the fourth most common cancer among both men and women, with nearly 1.09 million new cases and 0.55 million deaths each year worldwide [1]. The role of immune checkpoints (IC) in suppression of anti-tumor immune responses to various malignancies provided new targets for immunotherapy. Despite the clinical efficacy of current available immunotherapies in cancer patients, a large proportion of CRC patients fail to respond mainly due to immune-cell mediated resistance [2].

T-cell immunoglobulin and mucin domain containing 3 (TIM-3) is a member of mucin domain transmembrane protein family, which was initially recognized on CD4^+^ T helper 1 (Th1) and CD8^+^ T cytotoxic (Tc1) cells [3,4]. Later studies revealed that TIM-3 can also be expressed on other immune subsets, including T regulatory cells (Tregs) [5]. Reports showed that TIM-3 can activate the TCR signaling more likely towards exhaustion phenotypes with co-expression of other ICs in CRC patients [6,7]. Accumulating evidence suggests that expression of TIM-3 on tumor-infiltrating lymphocytes (TILs) has an indispensable role in tumor biology [8,9]. TIM-3 plays an important role in tumorigenesis and progression of prostate cancer and also has a potential prognostic value as a biomarker [10]. Moreover, TIM-3 serves as a potential mediator for tumor progression in CRC [9]. The characterization of TIM-3 in various human tumors including hepatocellular, cervical, colorectal, and ovarian carcinomas showed that TIM-3-expressing CD4^+^ TILs could represent the functional regulatory T cells with highly immunosuppressive characteristics [8]. Additionally, FoxP3^+^ Tregs co-expressing TIM-3 and PD-1, were reported to be highly immunosuppressive including a specialized subset of tissue Tregs in the breast tumor models [11]. The co-blockade of TIM-3 and PD-1 could downregulate genes including IL-10, CCL4, CCL5, PRF-1, IL-1R2, and RGS16, which are associated with TIM-3^+^ Treg suppressor functions [11]. However, the precise mechanism of TIM-3 and its effect on immune signaling cascade in the TME is still elusive.

In this study, we investigated the phenotypic and transcriptomic characteristics of tumor-infiltrating CD4^+^TIM-3^+^ T cells in CRC patients. We found that TIM-3 expression on CD4^+^ T cells was upregulated in TILs, compared with cells isolated from paired, adjacent normal colon tissues (NILs). Moreover, TIM-3^+^ cells in TILs show more Treg-signature markers including Forkhead box P3 (FoxP3), CD25, and Helios, and co-inhibitory molecules including programmed cell death-1 (PD-1), cytotoxic T-lymphocyte-associated antigen 4 (CTLA-4), and lymphocyte-activation gene 3 (LAG-3). These data suggest that TIM-3 expression on Tregs can contribute to a highly immunosuppressive tumor microenvironment, which may favor immune evasion and tumor progression. Our transcriptomic data showed that TIM-3^+^ TILs have potential roles in tumor metastasis, T-cell exhaustion, and tumorigenesis pathways. Additionally, Treg-related genes were upregulated on TIM-3^+^ T cells, compared with conventional CD25^+^ Treg. Altogether our data suggest that the upregulation of TIM-3 on CD4^+^ TILs could alter the cells to have more immunosuppressive, migratory, and exhausted phenotype, which contribute to tumor evasion and metastasis. Of note, this work lacks functional studies due to limited cell numbers following FACS sorting.

## 2. Results

### 2.1. TIM-3 Is Highly Upregulated on Tumor-Infiltrating T Cells in Colorectal Cancer

**Figure 1:**
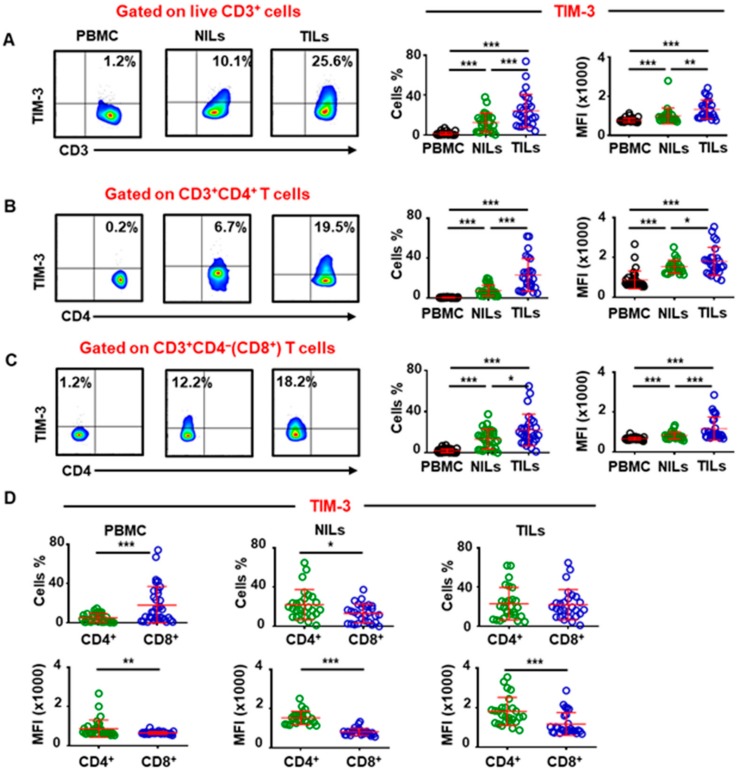
Comparison of T-cell immunoglobulin and mucin domain containing 3 (TIM-3^+^) T cells in peripheral blood mononuclear cells (PBMC), normal colon tissues (NILs), and tumor-infiltrating lymphocytes (TILs) of colorectal cancer (CRC) patients. Percentage and mean fluorescence intensity (MFI) of TIM-3^+^ T cells was analyzed by flow cytometry. Representative flow cytometric plots and scatter plots showing TIM-3 expression in PBMC, NILs, and TILs on CD3^+^ (**A**), CD3^+^CD4^+^ (**B**), and CD3^+^CD4^−^ (CD8^+^) T cells (**C**). Scatter plots show comparison of the percentage and MFI of TIM-3^+^ cells on CD3^+^CD4^+^ and CD3^+^CD4^−^ (CD8^+^) T cells in PBMC, NILs, and TILs (**D**). The *p* values are represented as follows; *** *p* <0.001, ** *p* < 0.01, * *p* < 0.05.

TIM-3 has been identified as a potential prognostic biomarker in various solid malignancies, including CRC, in which higher TIM-3 expression has been shown to be associated with decreased overall survival [12]. We investigated TIM-3 expression on CD3^+^, CD3^+^CD4^+^, and CD3^+^CD4^−^(CD8^+^) T cells in PBMC from 34 CRC patients, and NILs and TILs from 27 CRC patients using flow cytometry. Of note, the expression profile of proteins could be changed during processing of samples for flow cytometric analyses, which might not reflect similar *in vivo* expression profiles. Herein, we found that TIM-3 expression was significantly higher in TILs (24.2% ± 3.2%), compared with NILs (12.5% ± 1.8%) and PBMC (1.3% ± 0.3%) (Figure 1A). TIM-3 was expressed at very low levels on CD4^+^ T cells in circulation, compared to normal colon tissue but was highly expressed on CD4^+^ TILs (0.5% ± 0.1% vs. 7.5% ± 1.0% vs. 21.2% ± 3.2%, Figure 1B). This expression pattern was also observed on CD8^+^ T cells as TIM-3 was highly upregulated on CD8^+^ TILs compared to NILs and PBMC from CRC patients (22.2% ± 3.0% vs. 13.2% ± 1.3% vs. 1.8% ± 0.3%, Figure 1C). We then wanted to investigate the differences in TIM-3 expression on CD4^+^ and CD8^+^ T cells in circulation, normal tissue, and TME. We found that TIM-3 is expressed at higher levels on CD8^+^ T cells than CD4^+^ T cells in periphery (Figure 1D). In contrast, significantly lower TIM-3 expression was detected on CD8^+^ NILs than CD4^+^ NILs, while no difference was detected in TIM-3 expression on CD4^+^ and CD8^+^ TILs (Figure 1D). Previous reports have suggested TIM-3 expression on CD4^+^ and CD8^+^ T cells is associated with T-cell exhaustion and anergy [13]. Since we did not find any differences in TIM-3 expression on CD4^+^ and CD8^+^ TILs, we focused our investigations on CD4^+^ T cells to study the significance of TIM-3 expression on T cells/Tregs in the CRC TME.

### 2.2. CD4^+^TIM-3^+^ T Cells in the Tumor Microenvironment Have More Immunosuppressive Characteristics

**Figure 2:**
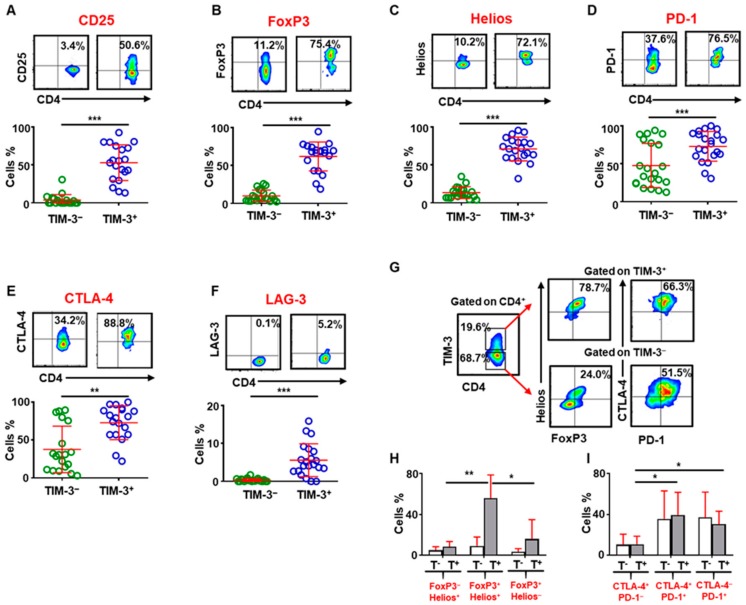
Analysis of the different surface markers expressed on TIM-3^+^ and TIM-3^−^ CD4^+^ TILs. Representative flow cytometric plots and scatter plots gated on CD4^+^ T cells show the percentage of CD25 (**A**), FoxP3 (**B**), Helios (**C**), PD-1 (**D**), CTLA-4 (**E**), and LAG-3 (**F**) expression in CD4^+^TIM-3^+^ and CD4^+^TIM-3^−^ TILs. Representative flow cytometric plots show FoxP3/Helios, and CTLA-4/PD-1 co-expression on CD4^+^TIM-3^+^ and CD4^+^TIM-3^−^ TILs (**G**). Bar plots show the percentage of FoxP3^+/−^Helios^+/−^ (**H**) and PD-1^+/−^CTLA-4^+/−^ (**I**) expression in CD4^+^TIM-3^−^ (T^−^) and CD4^+^TIM-3^+^ (T^+^) TILs. The *p* values are represented as follows; *** *p* <0.001, ** *p* < 0.01, * *p* < 0.05.

The immune landscape of CRC TME comprises of diverse populations that modulate anti-tumor responses. We and others have previously shown accumulation of immunosuppressive myeloid cells and Treg expressing multiple IC in CRC TME [14,15,16]. Moreover, previous studies have reported TIM-3 expression on dysfunctional T cells in various malignancies [17]. In this study, we found that CD4^+^TIM-3^+^ T cells within the CRC TME express CD25 and comprise mainly of FoxP3^+^ Treg that express high levels of Helios and also multiple IC, suggestive of highly suppressive and active phenotype. CD4^+^TIM-3^+^ T cells showed significantly higher CD25 (53.0% ± 5.3% vs. 3.8% ± 1.6%, Figure 2A) and FoxP3 expression (62% ± 4% vs. 10.1% ± 1.7%, Figure 2B) than CD4^+^TIM-3^−^ cells. Helios is a key transcription factor, which dictates the suppressive potential of FoxP3^+^ Treg by stabilizing FoxP3 [18]. We found significantly higher Helios expression on CD4^+^TIM-3^+^ cells than CD4^+^TIM-3^−^ cells (71.1% ± 3.5% vs. 13.6% ± 1.7%, Figure 2C). We also found elevated IC expression, including PD-1 (73.0% ± 4.7% vs. 47.8% ± 6.4%, Figure 2D), CTLA-4 (72.8% ± 5.1% vs. 37.7% ± 7.0%, Figure 2E), and LAG-3 (5.6% ± 0.9% vs. 0.3% ± 0.1%, Figure 2F) on CD4^+^TIM-3^+^ cells. Next, we compared FoxP3 and Helios co-expression to equate suppressive potentials of CD4^+^TIM-3^+/−^ subsets (Figure 2G). We found that the majority of CD4^+^TIM-3^+^ expresses both FoxP3 and Helios indicative of higher immunosuppressive potentials than CD4^+^TIM-3^−^ cells (Figure 2H). Accumulating evidence has shown that multiple IC are co-expressed on cancer-specific T cells that employ distinct signaling pathways [19]. We found that PD-1 and CTLA-4 are mainly co-expressed on CD4^+^ T cells but there are no significant differences in their co-expression within CD4^+^TIM-3^+/−^ cells in colorectal TME (Figure 2G,I).

In addition, we found that tumor-infiltrating CD25^+^ T cell express TIM-3 at significantly higher levels than CD25^−^ TILs and also co-express CTLA-4 and PD-1, but do not express LAG-3 (Supplementary Figure S1A,B). Additionally, the majority of CD4^+^CD25^+^ TILs isolated from CRC patients co-expressed FoxP3 and Helios (Supplementary Figure S1A,B). These cells, therefore, presented with highly suppressive phenotype, while high IC expression indicates their high activation states. We also found that the accumulation of TIM-3^+^ T cells in the CRC TME is higher than infiltration of CD25^+^ T cells (Supplementary Figure S1C).

### 2.3. Transcriptomic Profile of CD4^+^TIM-3^+^ TILs Reveals Their Potential Role in Tumor Proliferation and Metastasis

**Figure 3:**
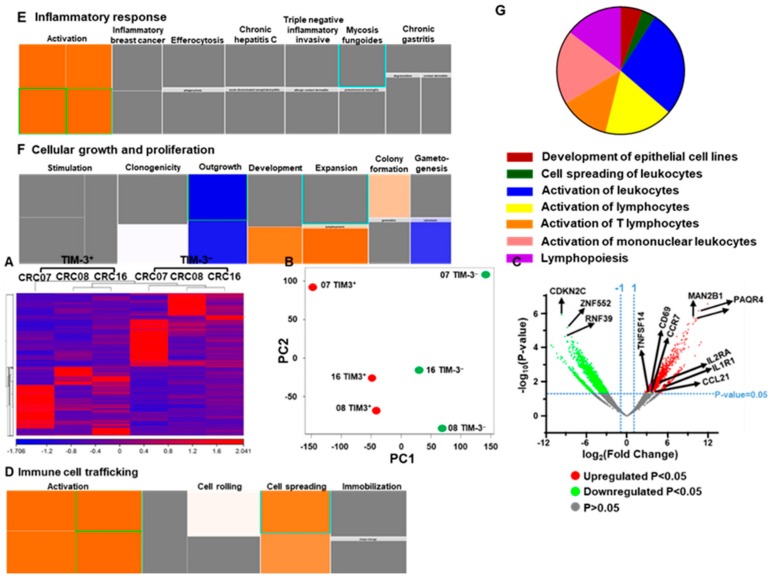
Differential gene expression of CD4^+^TIM-3^+^ and CD4^+^TIM-3^−^ TILs. Hierarchical clustering of three TIM-3^+^ and three TIM-3^−^ libraries (from patients CRC07, 08, and 16) on differentially expressed RNA transcripts from RNA-Sequencing data. Each column represents a sample and each row represents a transcript. Expression level of each gene in a single sample is depicted according to color scale (**A**). PCA plot shows the clustering of RNA transcriptome from of three TIM-3^+^ and TIM-3^−^ libraries (**B**). Volcano plot summarizes the expression rate (log2 Fold Change) on the *x*-axis and the statistical significance (negative log_10_-transformed *p* values) on the *y*-axis. The fold changes with significant *p* value (>0.05) of upregulated and downregulated genes are highlighted in red and green, respectively (**C**). Tree map (hierarchical heat map) depicting affected functional categories based on upregulated transcripts, immune cell trafficking (**D**), inflammatory response (**E**), and cellular growth and proliferation (**F**). Each individual colored rectangle is a particular biological function and the color range indicates its predicted activation state: increasing (orange) or decreasing (blue). Darker colors indicate absolute Z-scores. In this default view, the size of the rectangle is correlated with increased overlap significance. Top significantly upregulated migration and metastasis-related genes in TIM-3^+^ T cells (Z-score of >1.45) based on ingenuity pathways analysis (IPA) analyses are shown as a pie chart (**G**).

It has been shown that TIM-3 expression on CD4^+^ TILs may promote potential metastatic characteristics in cervical cancer TME [20]. We sorted CD4^+^TIM-3^+^ and CD4^+^TIM-3^−^ T cells from three CRC NT and TT samples (CRC07, 08, and 16, Supplementary Figure S2). Libraries were prepared and RNA-Sequencing was performed. This was done on few samples because of sample limitation and technical difficulty to generate libraries from very few cell numbers. Similarly, CTLA-4^+^ and CTLA-4^−^ Tregs from head and neck cancer patients were compared in very small number of samples [21]. We found that the hierarchical clustering of differentially expressed genes showed distinct cluster of TIM-3^+^ and TIM-3^−^ T cells (Figure 3A). A total of 1437 upregulated (>2-fold change, FC) and 1539 downregulated (>2-FC) transcripts were identified in TIM-3^+^, compared with TIM-3^−^ CD4^+^ T cells. The principal component analysis (PCA) from the transcripts per million (TPM) showed that the TIM-3^+^ and TIM-3^−^ from three patient samples were distributed close to each other, representing the significant differences in the overall gene expression (Figure 3B). The PCA plot also showed high variability in the expression of TIM-3 among the individuals, but it should not affect the downstream analyses due to the cluster separation of PC1 genes between TIM-3^+^ and TIM-3^−^ populations. The top 500 PC1 genes identified using PCA loading analyses are shown in Supplementary Table S1. The differential expression of significantly upregulated and downregulated genes is also represented as a volcano plot (Figure 3C). Canonical pathway analysis of both upregulated and downregulated genes showed that immune cell trafficking (Figure 3D), inflammatory response (Figure 3E), and proliferation (Figure 3F)-related genes were upregulated, while cell growth-related genes (Figure 3F) were downregulated in TIM-3^+^, compared with TIM-3^−^ cells. Interestingly, we found that signals involved in tumor migration and metastasis, including development of epithelial cells (6%), cell spreading of leukocytes (3%), activation of leukocytes (27%), activation of lymphocytes (17%), activation of T lymphocytes (13%), activation of mononuclear leukocytes (19%), and lymphopoiesis (15%) were upregulated with a Z-score of >1.45 in TIM-3^+^, compared with TIM-3^−^ cells (Figure 3G). We found that transcripts including EPHA3, ICOS, ITGB2, ITGA3, ANXA1, CD3E, BTK, IL24, CDN1, IL2RA, IKZF1, RAB29, TNFSF4, CD1D, AIRE, and CD69, which have potential roles in T cell activation and cellular migration were upregulated in TIM-3^+^, compared with TIM-3^−^ T cells (Figure 3). These data are in accordance with some reports that the overexpression of CD69 [22], ICOS [23], and EPHA3 [24] have crucial roles in migration and metastasis of CRC. Moreover, genes including IL2RA, CD3E, CD69, and ICOS which have critical roles in survival and lymphocyte activation were also upregulated in TIM-3^+^, compared with TIM-3^−^ T cells (Figure 3).

### 2.4. CD4^+^TIM-3^+^ T Cells Show an Exhausted Phenotype

**Figure 4:**
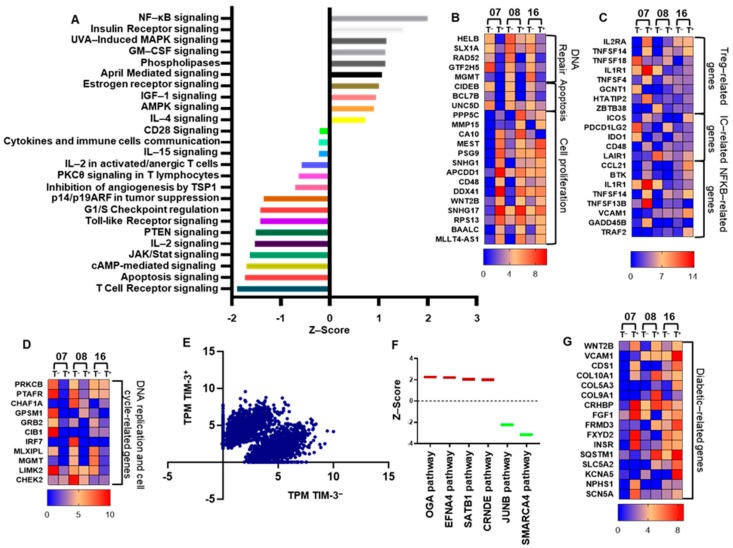
Transcriptomic profile of CD4^+^TIM-3^+^ and CD4^+^TIM-3^−^ TILs categorized by their functional characteristics. Top significantly affected (>−2 Z score < 2) canonical pathways based on IPA analysis. The horizontal bars denote the different pathways based on the Z-scores (**A**). Heat maps show the fold changes relative to the mean expression of DNA repair, apoptosis, and cell proliferation (**B**), Treg, IC, and NF-κB-related genes (**C**), DNA replication and cell cycle-related genes (**D**). Scatter plots show gene expression analyses by RNA-Sequencing in TIM-3^+^ and TIM-3^−^ T cells. X and Y axes represent TPM (Transcripts Per Million) of TIM-3^+^ and TIM-3^−^ T cells (**E**). Box plots show the Z-score of genes involved in tumor progression pathways of TIM-3^+^ T cells based on IPA analysis (**F**). Heat maps show the fold changes relative to the mean expression of diabetic-related genes (**G**).

The overexpression of TIM-3 on T cells in the TME can enhance T cell activation, leading to acceleration of series of pathways including NF-κB that drive T cells more likely toward an exhausted phenotype [6]. In addition, cAMP/PKC [25] and PTEN [26] signaling cascades can inhibit the activation of NF-κB transcription. Herein, we found that CD4^+^TIM-3^+^ cells upregulate exhausted T-cell pathways, compared with CD4^+^TIM-3^−^ T cells (Figure 4A). T-cell exhaustion and carcinogenesis signaling cascades including NF-κB, IL-4, AMPK, IGF-1, April, Estrogen receptor, GM-CSF, and insulin receptor were upregulated in TIM-3^+^ T cells (Figure 4A). Notably, the signaling pathways including TCR receptor, cAMP, JAK/STAT, Toll-like receptor, PKC, CD28, cytokine-immune cell communication, IL2 activation, which are indispensable for maintaining T cell-mediated immune homeostasis, were downregulated in TIM-3^+^ cells (Figure 4A). These data demonstrate that TIM-3 expression can downregulate PTEN/PKCθ and upregulate NF-κB signaling, which lead to T-cell exhaustion. Interestingly, we found that DNA replication-related genes including HELB, SLX1, RAD52, GTF2H5, and MGMT, cellular apoptosis-related genes including CIDEB, BCL7B, and UNC5D, and DNA replication/cell cycle-related genes including PRKCB, PTAFR, CHAF1A, GPSM1, GRB2, CIB1, IRF7, MLXIPL, MGMT, LIMK2, and CHEK2 were significantly downregulated in TIM-3^+^, compared with TIM-3^−^ T cells (Figure 4B,D, *p* value < 0.01). Additionally, cell proliferation (PPP5C, MMP15, CA10, MEST, PSG9, SNHG1, APCDD1, CD48, DDX41, WNT2B, SNHG17, RPS13, BAALC, and MLLT4-AS1) and NF-κB (CCL21, BTK, IL1R1, TNFSF14, TNFSF13B, VCAM1, GADD45B, and TRAF2) and Treg (IL2RA, TNFSF14, IL1R1, TNFSF4, GCNT1, HTATIP2, and ZTB38)-related genes were significantly upregulated in TIM-3^+^, compared with TIM-3^−^ (Figure 4B,C). Next, we checked the expression of other IC/ligands. We found that ICOS, TNFSF4 (OX40), CD48, and TNFSF14 were upregulated and TNFSF18 (GITR), PDCD1LG2, IDO1, LAIR1 were downregulated in TIM-3^+^, compared with TIM-3^−^ T cells (Figure 4C, *p* value < 0.01). The differential expression of gene clustering scatter plots shows a clear separation of upregulated and downregulated genes in TIM3^+^ T cells (Figure 4E). Moreover, the carcinogenesis and tumor metastasis-related signaling pathways including the upregulation of O-GlcNAcylation (OGA), Ephrin A4 (EFNA4), special AT-rich sequence-binding protein 1 (SATB1), and long non-coding RNA colorectal neoplasia differentially expressed (CRNDE), and downregulation of JUNB, SMARCA4 signaling pathways were observed in TIM-3^+^, compared with TIM-3^−^ T cell (Figure 4F).

It has been reported that TIM-3 can serve as a biological marker for type 2 diabetes mellitus (T2DM) [27]. The clinicopathological data of patients that we selected for RNA-Sequencing showed they all presented with T2DM (Table 1). Our transcriptomic analyses showed that diabetic-related genes including WNT2B, VCAM1, CDS1, COL10A1, COL5A3, COL9A1, CRHBP, FGF1, FRMD3, FXYD2, INSR, SQSTM1, SLC5A2, KCNA5, NPHS1, and SCN5A were upregulated in TIM-3^+^, compared with TIM-3^−^ T cells (Figure 4G, *p* value < 0.01).

### 2.5. CD4^+^TIM-3^+^ TILs Upregulate Treg-, Cell Migration-, and Tumor Metastasis-Related Genes, Compared with Conventional CD4^+^CD25^+^ Tregs

**Figure 5:**
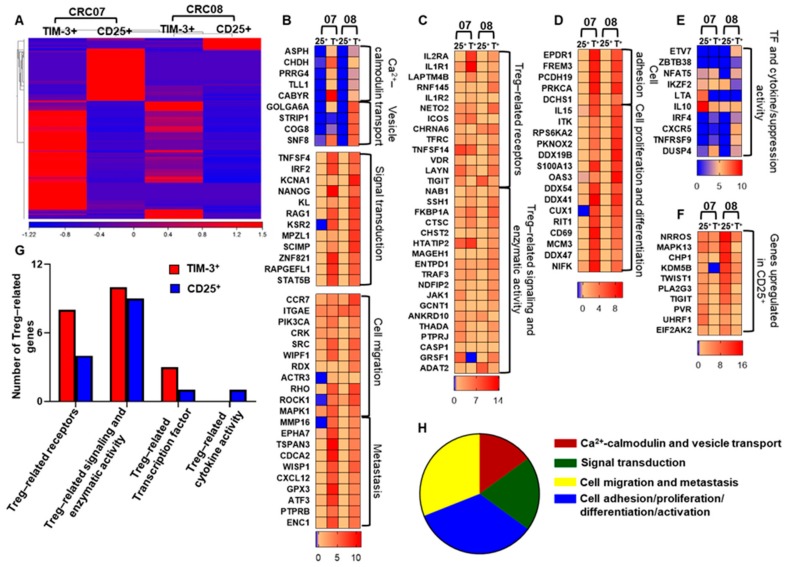
Transcriptomic profiles of TIM-3^+^ and CD25^+^ T cells. Hierarchical clustering of two TIM-3^+^ and two CD25^+^ libraries (patient IDs: CRC07 and 08) on differentially expressed RNA transcripts from RNA-Sequencing data. Each column represents a sample and each row represents a transcript. Expression level of each gene in a single sample is depicted according to color scale (**A**). Heat maps show the fold changes relative to the mean expression of calcium-calmodulin, vesicle transport, signal transduction, cell migration, and metastasis (**B**), Treg-related receptors and signaling and enzymatic activity genes (**C**), cell adhesion, proliferation, and differentiation (**D**). Transcription factor and cytokine/suppression activity (**E**) related genes and some of the selected genes upregulated in CD25^+^ T cells (**F**). The overall difference in the number of genes related to Tregs are shown as a bar plot (**G**). Top significantly upregulated migration and metastasis-related genes in TIM-3^+^, compared with CD25^+^ T cells (Z-score of >1.5) based on IPA analysis are shown as a pie chart (**H**).

Next, we compared the transcriptomic profile of CD4^+^TIM-3^+^ T cells with conventional CD4^+^CD25^+^ Tregs. We sorted CD4^+^TIM-3^+^ and CD4^+^CD25^+^ T cells from three CRC NT and TT samples. We found that about half of the TIM-3^+^ cells were CD25^−^ population (Supplementary Figure S3). Library preparation and subsequent RNA-Sequencing were performed for CD4^+^TIM-3^+^ and CD4^+^CD25^+^ T cells from two TT samples (CRC07 and 08). Here, we found a distinct cluster of differentially expressed genes in TIM-3^+^ and CD25^+^ T cells (Figure 5A). Next, we found that genes related to cell migration including ACTR3, CCR7, ITGAE, PIK3CA, CRC, SRC, WIPF1, RDX, RHO, ROCK1, and MAPK1 [28] were upregulated in CD4^+^TIM3^+^ TILs (Figure 5B, bottom heat map). Additionally, CD4^+^TIM3^+^ TILs also upregulated tumor metastasis-related genes, including MMP16, EPHA7, TSPAN3, CDCA2, WISP1, CXCL12, GPX3, ATF3, PTPRB, and EC1 (Figure 5B, bottom heat map, *p* value < 0.01). Genes including calcium-calmodulin signaling (ASPH, CHDH, PRRG4, TLL1, and CABYR), vesicle transport (GOLGA6A, STRIP1, COG8, and SNF8), signal transduction (TNFSF4, IRF2, KCNA1, NANOG, KL, RAG1, KSR2, MPZL1, SCIMP, ZNF821, RAPGEFL1, and STAT5B), cell migration (PLAT, RLTPR, TPTEP1, SNX5, CHST3, PRKCE, CRMP1, and SYNE3) and tumor metastasis (MMP16, EPHA7, TSPAN3, CDCA2, WISP1, CXCL12, GPX3, ATF3, PTPRB, and ENC1) were significantly upregulated in TIM-3^+^, compared with CD25^+^ T cells (Figure 5B,H, *p* value < 0.01). The hierarchy of the percentages of upregulated gene categories are as follows; cell adhesion/proliferation/differentiation/activation (34%) > cell migration and metastasis (31%) > signal transduction (20%) > Ca2^+^ calmodulin and vesicle transport (15%) (Figure 5H). Additionally, we found that Treg-related genes [29] including IL2RA, IL1R1, LAPTM4B, RNF145, IL1R2, NETO2, ICOS, CHRNA6, TFRC, TNFSF14, VDR, LAYN, and TIGIT, involved in receptor signaling (Figure 5C,G); NAB1, SSH1, FKBP1A, CTSC, CHST2, HTATIP2, MAGEH1, ENTPD1, TRAF3, NDFIP2, JAK1, GCNT1, ANKRD10, THADA, and PTPRJ, involved in enzymatic activity (Figure 5C,G); transcription factor and suppression activity-related genes including ETV7, ZBTB38, NFAT2, IKZF2, LTA, IRF4, CXCR5, TNFRSF9, and DUSP4, were significantly upregulated, and IL10 and IKZF2 (Figure 5E,G) were downregulated in TIM-3^+^, compared with CD25^+^ TILs. Furthermore, genes related to cell-cell adhesion (EPDR1, FREM3, PCDH19, PRKCA, and DCHS1) and cell proliferation and differentiation (some of the genes including IL15, ITK, OAS3, DDX54, DDX41, CUX1, CD69, and NIFK), were upregulated in TIM-3^+^ cells (Figure 5D,H, *p* value < 0.01). These data suggest that CD4^+^TIM-3^+^ TILs may contribute more than conventional CD4^+^CD25^+^ Tregs to the tumor evasion and metastasis.

It has been shown that TIGIT expression on Tregs within the TME shows an exhausted phenotype with more activated and suppressive characteristics, compared with conventional CD25^+^ Tregs [30]. We also found that TIGIT and its ligand, PVR, were significantly upregulated in CD25^+^ compared with TIM-3^+^ T cells (Figure 5F, *p* value < 0.01). Further functional studies are warranted to identify the role of TIGIT in the CRC TME.

## 3. Discussion

Accumulating evidences from in vivo studies have shown that TIM-3 blockade enhances anti-tumor immunity and suppresses tumor growth [11,31]. Therefore, TIM-3 could be considered as a potential candidate for IC inhibition due to the remarkable success of other IC inhibitors in treating various cancers.

We found elevated TIM-3 expression on CD3^+^, CD4^+^, and CD8^+^ T cells in the colorectal TME. Studies showed that TIM-3 and PD-1 co-expression on CD8^+^ T cells exhibits more exhausted phenotypes, as defined by failure in cytokine secretion including interferon-γ (IFN-γ), tumor necrosis factor-α (TNF-α), and interleukin-2 (IL-2) in CRC patients [32]. Additionally, co-blockade of TIM-3 and PD-1 reverses this induced T-cell exhaustion [32]. Our results showed that TIM-3 is expressed at higher levels on CD8^+^ T cells in circulation of CRC patients, but it is expressed at significantly lower levels than CD4^+^ T cells in normal colon. However, we did not find any difference in TIM-3 expression between CD4^+^ and CD8^+^ TILs (Figure 1D).

Accumulation of FoxP3^+^ Treg in the TME of various malignancies, favoring tumor progression is widely reported [33]. We found that CD4^+^TIM-3^+^ cells in the TME comprise mainly of CD25^+^FoxP3^+^ Treg that express Helios and also co-express other ICs, PD-1, and CTLA-4 but not LAG-3. Helios is a key transcription factor, identified as a marker of activated Treg that provides stability to their suppressive function [18,34]. Targeting Helios leads to conversion of Treg to effector cell and increased anti-tumor responses in tumor-bearing mice [35]. The presence of highly suppressive Tregs with multiple IC expression in the TME contribute towards tumor immune evasion mechanisms through affecting immune effector signaling pathways; Treg suppress effector T cell functions and impaired cytokine release from IC-expressing T cells are capable of neutralizing the impact of tumor-specific T cells. Studies have shown that there is an increase in the ratio of effector T cells to Tregs within the TME with IC blockade [4]. Of note, we have recently reported that CD4^+^TIM-3^+^ T cells in circulation can promote proliferation of responder T cells, which was not affected by PD-1 blockade in vitro [36].

The expression of TIM-3 at higher levels in colorectal cancer tissues is significantly associated with tumor size and tumor-node/distant metastasis [9]. Knockdown of TIM-3 in CRC cell lines HCT116 and HT-29 significantly reduced cell proliferation rates compared with controls [9]. TIM-3 also promotes inflammation through the synergetic action with TLR signaling in immune cells [9]. Our network analysis showed that immune cell trafficking/inflammatory response-mediated genes were upregulated in TIM-3^+^ T cells (Figure 3 and Figure S4A). Development of CRC is through an array of genetic modifications that can convert normal colon epithelium to adenocarcinoma including proliferation, migration, survival, and inflammation. Here, we showed that the genes involved in triggering of tumor metastatic pathways were higher in TIM-3^+^ T cells (Figure 3F). Altogether, our data suggest that TIM-3 expression on CD4^+^ TILs could trigger pathways involved in tumor metastasis.

TIM-3 expression on both CD4^+^ and CD8^+^ T cells is directly related to T-cell exhaustion in CRC patients, controlled by various TCR signaling pathways [6,7]. The ectopic TIM-3 expression on T cells can upregulate NF-κB through an array of signaling cascade including NFAT/AP-1/PI3/AKT/cytokine production, ultimately triggers the anti-apoptosis and T-cell exhaustion pathways [37,38]. Our results show that the four indispensable signaling pathways to keep immune system in check; TCR [39], IL2 activation [40], JAK/STAT [41], and cAMP-mediated signaling [42] were downregulated in TIM-3^+^ T cells (Figure 4A). These dysregulations could create an immune-subversive environment for tumor cells to survive. Moreover, the distinguished downregulation of PTEN signaling could be due to the upregulation of NF-κB-mediated signaling (Figure 4A) and may contribute to T-cell exhaustion. These observations could help us to understand the role of TIM-3 in CRC and immune exhaustion.

Next, we showed that four signaling pathways including OGA, EFNA4, SATB1, and CRNDE were overexpressed in TIM-3^+^ T cells (Figure 4F). OGA has been shown to play a significant role in the progression of CRC by altering the proliferation/invasion-related transcripts through NF-κB-mediated signaling [43]. Our results also showed that NF-κB signaling and related genes were upregulated in TIM3^+^ T cells (Figure 4A and C). Additionally, Ephrin receptors including EFNA4 belong to tyrosine kinase family [44], SATB1 [45] and CRNDE [46] have been shown to be elevated in CRC and other cancers, which lead to poor prognosis and metastasis. Furthermore, tumor suppressor gene pathways including JUNB [47] and SMARCA4 [40], which inhibit proliferation and metastasis were significantly downregulated in TIM-3^+^ T cells. In addition to tumor suppressor genes, we also found that DNA repair/replication/cell cycle and apoptosis-related genes were also downregulated in TIM-3^+^ T cells. Notably, cell proliferation-related genes were upregulated in TIM-3^+^ T cells. Our gene expression, cellular development, and cancer network analysis also confirmed the role of TIM-3^+^ T cells in cancer development (Supplementary Figure S4B). Altogether, our data suggest that the upregulation of NF-κB-mediated transcription factors may prevent apoptosis by generating a negative regulatory loop with PTEN and favors proliferation and migration of TIM-3^+^ T cells.

A report showed that expression of TIM-3 was higher in both CD4^+^ and CD8^+^ T cells and lower in CD14^+^ monocytes in T2DM patients [27]. A meta-analysis report showed that diabetes has negative effects on survival of CRC patients [48]. Our data suggest that more diabetes-related genes were upregulated in TIM-3^+^, compared with TIM-3^−^ T cells (Figure 4G). Out of all genes, WNT2B, which was upregulated in TIM-3^+^ T cells have already been reported to play a dynamic role in the pathogenesis of T2DM [49]. It has been reported that TIM-3 expression was higher in PBMC of T2DM patients, compared with healthy donors [27]. In T2DM patients, TIM-3 was mainly expressed on CD4^+^, CD8^+^, and monocytes [27]. The correlation analyses showed that the expression of TIM-3 on CD4^+^ and CD8^+^ T cells was higher in patients with increased fasting plasma glucose [27]. However, the potential role of TIM-3 in T2DM remains to be elucidated. Of note, we performed RNA-Seq on cells isolated from T2DM patients and it would be interesting to compare the transcriptomic expression of CD4^+^TIM-3^+^ and CD4^+^TIM-3^−^ subpopulations from T2DM and non-T2DM patients.

Accumulating evidence suggests that TIM-3 is expressed not only on effector T cells, but also in tumor-infiltrating Tregs in multiple tumor types [8,50]. The increased expression of TIM 3 in tumor-infiltrating Tregs are also associated with poor survival [8,9]. Here we found that in CRC TME, TIM-3^+^ Tregs have higher expression of FoxP3, CD25, Helios, CCR7, and also co-inhibitory molecules including PD-1, CTLA4, and TIM-3 (Figure 2 and Figure S3). Furthermore, we found that Treg-related genes were also upregulated in TIM-3^+^ Tregs, compared with CD25^+^ Tregs (Figure 5G). We validated our results by investigating CD25 and CCR7 expression as activation markers on TIM-3^+^ T cells from 14 CRC patient samples in addition to the samples used for RNA-Sequencing (CRC07 and 08). We found that TIM-3^+^ T cells express CD25 and CCR7 by flow cytometry (Supplementary Figure S3A–C) and through transcriptomic data (Supplementary Figure S3D).

Additionally, our transcriptomic profiling of CD4^+^TIM3^+^ T cells and CD4^+^CD25^+^ Tregs showed that CD4^+^TIM-3^+^ T cells show more migration features, compared with conventional CD4^+^CD25^+^ (Figure 5H). Furthermore, the Treg suppressive function-related genes including IL-10 and IKZF2 were significantly downregulated in CD4^+^TIM3^+^ T cells, compared with CD4^+^CD25^+^ Tregs. These results indicate that TIM-3^+^ T cells may implement alternative pathways, other than IL-10, for their suppressive function. Taken together, our data suggest that that TIM-3 expression on Tregs in the CRC TME may modify conventional CD25^+^ Tregs to have more suppressive characteristics. However, transcriptomic studies are recommended using CD25^+^TIM3^+^ and CD25^+^TIM3^−^ Tregs to further elucidate the precise role of TIM -3 expression on CD25^+^ Tregs.

## 4. Materials and Methods

### 4.1. Sample Collection and Storage

Peripheral blood samples were collected in EDTA tubes from 34 colorectal cancer (CRC) patients. Additionally, out of the 34 patients, tumor tissues (TT) and paired, adjacent non-cancerous normal colon tissues (NT) were obtained from 27 patients who underwent surgery at Hamad Medical Corporation, Doha, Qatar. All patients included in the study were treatment-naïve prior to surgery and provided written informed consent prior to sample collection. Table 1 shows the clinical and pathological characteristics of all participating patients. All experiments were performed in accordance with relevant guidelines and regulations. This study was executed under ethical approvals from Hamad Medical Corporation, Doha, Qatar (Protocol no. MRC-02-18-012) and Qatar Biomedical Research Institute, Doha, Qatar (Protocol no. 2018-018).

Peripheral blood mononuclear cells (PBMC) were isolated from fresh blood by density-gradient centrifugation using Histopaque-1077 (Sigma-Aldrich, St. Louis, MO, USA). PBMC were frozen in freezing media ((50% FBS), 40% RPMI 1640 media and 10% DMSO)) at a density of 5 million cells per 1 mL in cryovials to be used in batches for subsequent analyses. Tissue specimens were also stored in freezing media to maintain viability of cells for subsequent analyses.

### 4.2. Cell Dissociation

Cells were isolated from TT by mechanical disaggregation. Briefly, tissues frozen in freezing media were thawed and washed with phosphate-buffered saline (PBS) and then mechanically cut into small pieces (≈2–4 mm) using a surgical scalpel. Further disaggregation was performed on gentleMACS dissociator (Miltenyi Biotech, Bergisch Gladbach, Germany) without using any enzymes. The cell suspension was then passed through a 100 µM cell strainer to remove debris and aggregates. The single cell suspension was washed with PBS and stained for flow cytometric analysis and FACS sorting.

### 4.3. Multi-Parametric Flow Cytometry

PBMC and cells isolated from tissues were washed with PBS and resuspended in 100 µL flow cytometry staining buffer (PBS with 1% FCS and 0.1% sodium azide). Fc receptors (FcR) were first blocked using FcR Blocker (Miltenyi Biotec). Fixable Viability Dye eFluor 780 (eBioscience, San Diego, CA, USA) was added to gate live cells only. Cells were then stained with cell surface antibodies against CD3-Alexa Fluor 700 (clone UCHT-1; BD Biosciences, Oxford, UK), CD4-phycoerythrin (clone RPA-T4; BD Biosciences), CD25-Brilliant Violet 650 (clone BC96; BioLegend, San Diego, CA, USA), PD-1-PE/Dazzle^TM^ 594 (clone EH12.2H7; BioLegend), LAG-3-Brilliant Violet 421 (clone T47-530; BD Biosciences), and TIM-3-Brilliant Violet 711 (clone 7D3; BD Biosciences) or chemokine receptor 7 (CCR7)-Brilliant Violet 711 (clone 2-L1-A; BD Biosciences), and incubated at 4 °C for 30 min. Cells were then washed twice with flow cytometry staining buffer.

Cell sorting was performed using cell surface markers and 7AAD viability staining solution (eBioscience) was used to gate live cells. Cells were resuspended in Pre-Sort buffer (BD Biosciences) for sorting.

For intracellular staining, cells were incubated at 4 °C for 45 min in fixation/permeabilization buffer (eBioscience). Cells were then washed twice with permeabilization wash buffer (eBioscience). Mouse serum (Sigma-Aldrich) and rat serum (Sigma-Aldrich) were added to block for 10 min at 4 °C. Intracellular antibodies including CTLA-4-PerCp-eFluor 710 (clone 14D3; eBioscience), FoxP3-phycoerythrin cyanin 7 (PE/Cy7) (clone PCH101; eBioscience), and Helios-Fluorescein Isothiocyanate (FITC) (clone 22F6; BioLegend) were added and cells incubated for another 30 min at 4 °C. Cells were then washed twice with permeabilization wash buffer (eBioscience) and resuspended in flow cytometry staining buffer.

All data were acquired on a BD LSRFortessa X-20 flow cytometer and cell sorting was performed on BD FACSAria III SORP cell sorter, using BD FACSDiva software (BD Biosciences). Applicable measures were taken to ensure minimal sorter-induced cell stress (SICS). Data analyses were performed on FlowJo V10 software (FlowJo, Ashland, OH, USA).

### 4.4. Library Preparation

A total of 1000 sorted pure CD4^+^TIM-3^+^, CD4^+^TIM-3^−^, and CD4^+^CD25^+^ T cells from TT were used to generate cDNA libraries for RNA-Sequencing using QIAseq FX Single Cell RNA Library Kit (Qiagen, Hilden, Germany) following the manufacturer’s instructions. Briefly, sorted cells were spun down and lysed immediately. The gDNA was then removed by using gDNA Wipeout buffer (Qiagen). The gDNA-removed lysates were used to generate double-stranded DNA, which were subsequently amplified using REPLI-g sc SensiPhi DNA polymerase. Small fractions of amplified products were cleaned using PureLink PCR Purification Kit (Thermo Fisher Scientific, Waltham, MA, USA) and quality checked using Agilent High Sensitivity DNA Kit (Agilent Technologies, Santa Clara, CA, USA). The quality passed DNA (>2000 bp) were quantified using Qubit dsDNA HS assay kit (Invitrogen, Carlsbad, CA, USA). A total of 500 ng to 1 µg DNA was enzymatically fragmented and ligated using paired adaptors. DNA was further purified using Agencourt AMPure XP beads (Beckman Coulter, Brea, CA, USA). The yield and size distribution of libraries (500–1000 bp) were determined using Qubit dsDNA HS assay kit (Invitrogen) and Agilent High Sensitivity DNA Kit (Invitrogen).

### 4.5. RNA-Sequencing Data Analyses

Pair end reads were aligned to the hg19 human reference genome in CLC Genomics Workbench 12 (Qiagen). The abundance of the expression of transcripts was measured as the score of RPKM (Reads Per Kilobase Million) mapped reads in CLC Genomics Workbench 12. Abundance data were subsequently subjected to differential gene expression using 2.0-fold change and <0.05 *p* value cut-off.

### 4.6. Gene Set Enrichment Analyses and Modeling of Gene Interactions

Differential gene expression profiles were imported into the ingenuity pathways analysis (IPA) software 8.7 (Ingenuity Systems; www.ingenuity.com/) to obtain functional regulatory networks and canonical pathways using upstream regulator analysis (URA), downstream effects analysis (DEA), mechanistic networks (MN), and causal network analysis (CNA) prediction algorithms. IPA uses precise database to paradigm functional regulatory networks from a list of individual genes and determines a statistical score for each network according to the fit of the network to the set of focus genes. The score is the negative log of P and denotes the likelihood of the focus genes in the network being found together by chance. The biological functions assigned to each network are ranked according to the significance of that biological function to the network [51,52].

### 4.7. Statistical Analyses

Statistical analyses were performed using GraphPad Prism 8 software (GraphPad Software, San Diego, CA, USA). One-way Anova test was performed to check for statistical significance in grouped analyses. Unpaired *t*-tests were performed on samples that passed the Shapiro–Wilk normality test and Mann–Whitney tests were performed for samples that did not show normal distribution. A *p* value of > 0.05 was considered statistically non-significant. The *p* values are represented as follows; *** *p* < 0.001, ** *p* < 0.01, * *p* < 0.05. Data are presented as mean ± standard deviation (SD).

## 5. Conclusions

**Figure 6:**
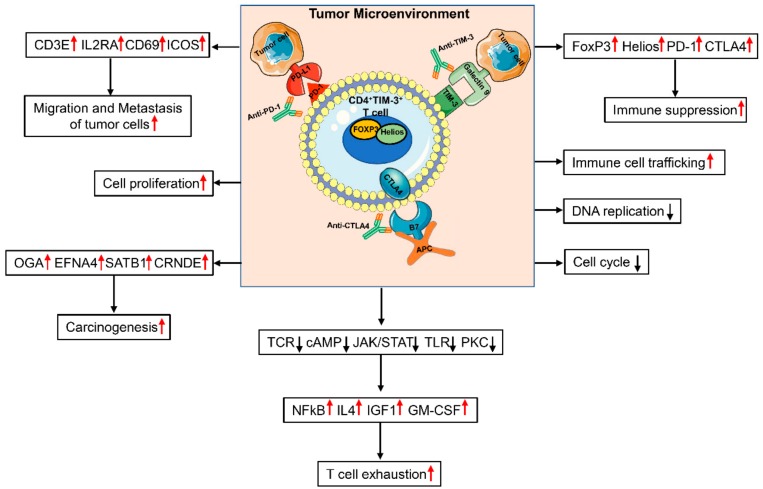
Schematic diagram summarizes genes/functions that are upregulated or downregulated in CD4^+^TIM-3^+^ T cells in the colorectal tumor microenvironment. In the colorectal TME, CD4^+^TIM-3^+^ cells co-express genes including FoxP3, Helios, PD-1, and CTLA-4 to suppress anti-tumor immune responses. Our study showed that DNA replication and cell cycle genes were downregulated, while genes related to immune suppression, cell proliferation, T-cell exhaustion, carcinogenesis, migration, and metastasis were upregulated in CD4^+^TIM-3^+^, compared with CD4^+^TIM-3^−^. Individual or combinational antibody therapies using anti-TIM-3, anti-PD-1, and anti-CTLA4 may target highly immunosuppressive TIM-3^+^ TILs to elicit anti-tumor responses in CRC.

Our data show that TIM-3 expression in the CRC TME is indicative of T-cell exhaustion and promotion of tumor metastasis. Accurate characterization of TIM-3^+^ T cells should enable targeting them in more specific approaches to enhance anti-tumor immunity and improve clinical responses. The overall findings from our study are summarized in Figure 6. This study gives an insight into the role of TIM-3 expression in the CRC TME. Clearly, this study lacks functional data due to limitations of low TIM-3 expression in CD4^+^ T cells in PBMC. Additionally, although TIM-3 expression was high in CD4^+^ TILs, the overall TIL numbers were very low to perform additional functional assays (Figure 1B).

## Figures and Tables

**Table 1:** Characteristic features of study populations.

	CRC Patients
**Number**	34 ^♦^ (27) * (3) ** (2) ***
**Age** (Median)	62 (31–96) †
**Gender** (Male:Female)	24:10
**TNM stage**	
I	4 ^♦^ (1) *
II	11 ^♦^ (10) * (2) ** (1) ***
III	16 ^♦^ (13) * (1) ** (1) ***
IV	3 ^♦^ (3) *
**Histological grade**	
G2—Moderately differentiated	All samples
**T2DM**	10 ^♦^ (8) * (3) ** (2) ***
**Hypertension**	16 ^♦^ (11) * (1) **

CRC; colorectal cancer. T2DM; type 2 diabetes mellitus. ^♦^ Samples used for investigating circulating immune cells. * Samples used for investigating tissue-infiltrating immune cells. ** Samples used for RNA-Sequencing analyses from CD4^+^TIM-3^+/−^ sorted tumor-infiltrating lymphocytes (TILs). *** Samples used for RNA-Sequencing analyses from CD4^+^CD25^+/−^ sorted TILs. † Data shown represent median (range).

## Supplementary Materials

The following are available online at https://www.mdpi.com/2076-393X/8/1/71/s1, Figure S1: Analysis of different surface markers in CD4+CD25+ and CD4+CD25− of the two samples used for RNA-Sequencing, Figure S2: Sorting strategy of TIM-3+ and TIM-3− T cells used for RNA-Sequencing, Figure S3: Validation of RNA-Sequencing data, Figure S4: Network analyses of differentially-expressed genes in CD4+TIM-3+ and CD4+TIM-3− TILs, Supplementary Table S1: PCA loading analysis TIM-3+ vs. TIM-3−.
