# Supplementary material for: Transcriptomic Profiling of Tumor-Infiltrating CD4+TIM-3+ T Cells Reveals Their Suppressive, Exhausted, and Metastatic Characteristics in Colorectal Cancer Patients
Source: Vaccines (Basel). 2020 Feb 6;8(1):71. doi: 10.3390/vaccines8010071 (PMC7157206; doi:10.3390/vaccines8010071)
Supplement: Supplementary file 1 [file vaccines-08-00071-s001.pdf]

Supplementary material

Transcriptomic Profiling of Tumor-Infiltrating CD4⁺TIM-3⁺ T Cells Reveals their Suppressive, Exhausted and Metastatic Characteristics in Colorectal Cancer Patients

Varun Sasidharan Nair, Salman M Toor, Rowaida Z Taha, Ayman A Ahmed, Mohamed A Kurer, Khaled Murshed, Madiha E Soofi, Khalid Ouararhni, Nehad M. Alajez, Mohamed Abu Nada and Eyad Elkord

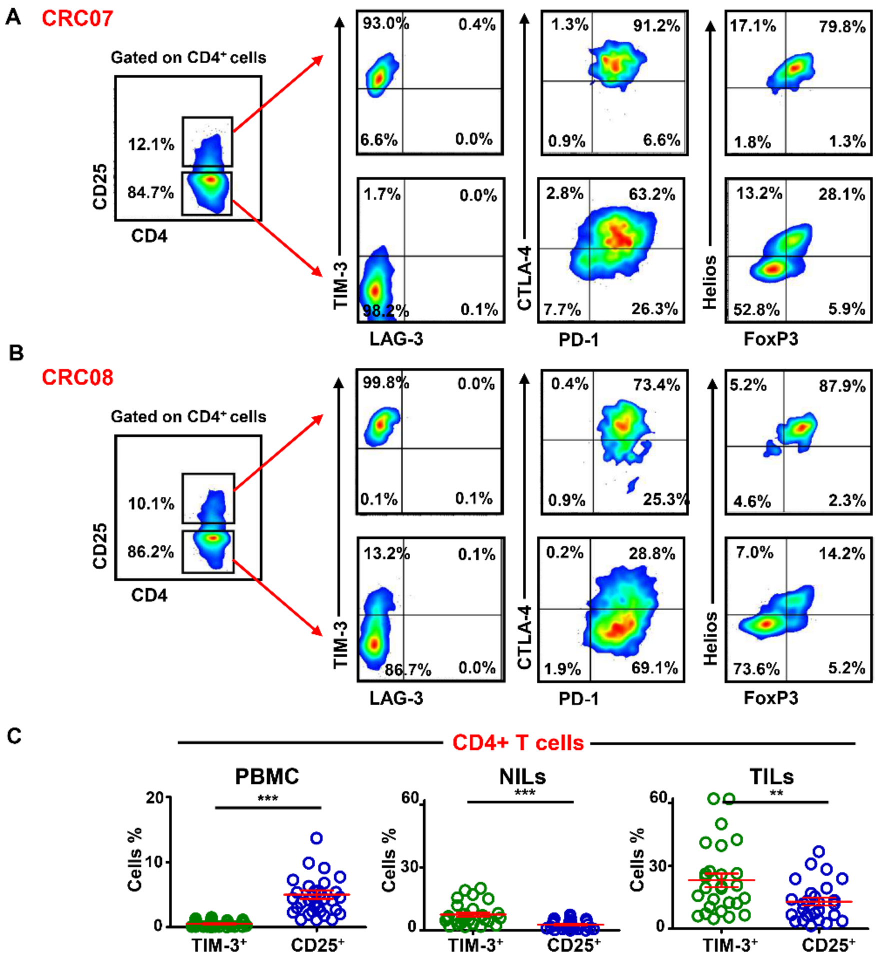

Figure S1. Analysis of expression of different surface markers on CD4⁺CD25⁺ and CD4⁺CD25⁻ of the two samples used for RNA-Sequencing. Flow cytometric plots show the percentage of TIM-3/LAG-3, CTLA-4/PD-1 and Helios/FoxP3 in CD4⁺CD25⁺ and CD4⁺CD25⁻ T cell populations of CRC07 (A) and CRC08 (B). Scatter plots comparing the overall percentage of TIM-3⁺ and CD25⁺, gated on CD4⁺ T cells in PBMC, NILs and TILs (C).

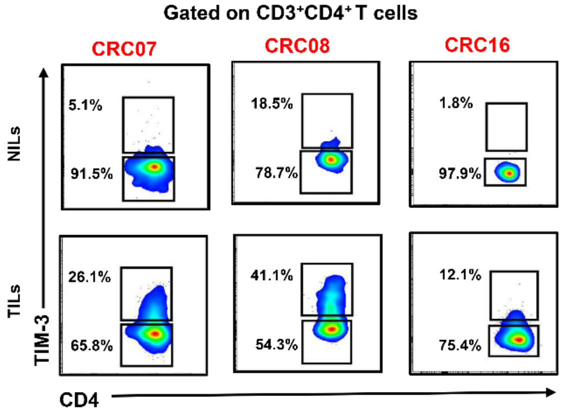

Figure S2. Sorting strategy of TIM-3^{+} and TIM-3^{-} T cells used for RNA-Sequencing. Flow cytometric plots show the percentage of TIM-3^{+} and TIM-3^{-} gated on CD3^{+}CD4^{+} T cells of CRC07, CRC08 and CRC16 used for RNA-Sequencing.

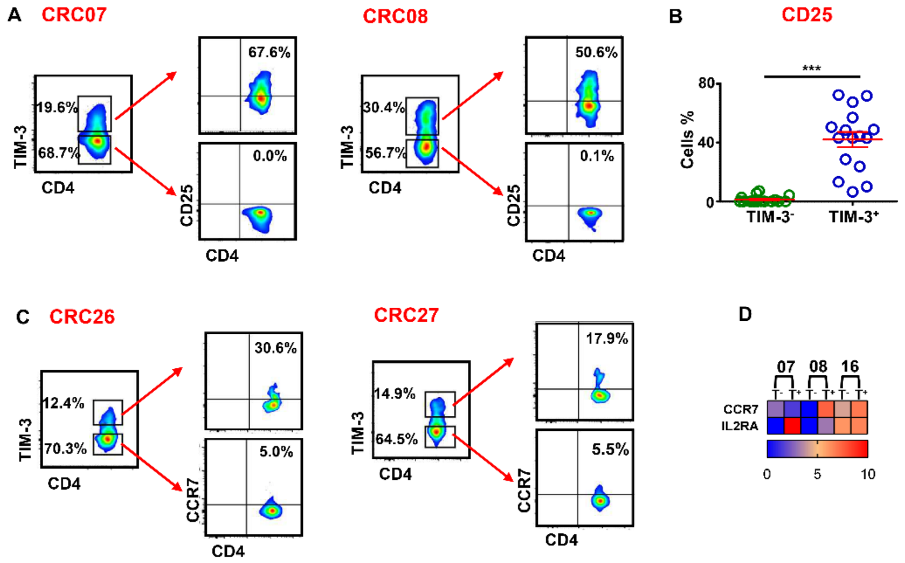

Figure S3. Validation of RNA-Sequencing data. The RNA-Sequencing data were validated by selecting two genes (CD25 and CCR7) that showed significant upregulation in TIM-3^{+} compared with TIM-3^{-} T cells in three patients. Representative flow cytometric plots show the percentage of CD25^{+} in CD4^{+}TIM-3$^{+/-}$ T cells of CRC07 and CRC08 (A). Scatter plot shows the differences in CD25 expression between CD4^{+}TIM-3^{+} and CD4^{+}TIM-3^{-} TILs in 16 samples (B). Flow cytometric plots show the expression of CCR7 in CD4^{+}TIM-3$^{+/-}$ T cells from CRC26 and CRC27 (C). Heat map shows the fold changes relative to the mean expression of CCR7 and IL2RA (CD25) in the three patients (CRC07, 08 & 16, D). Expression level of each gene in a single sample is depicted according to color scale.

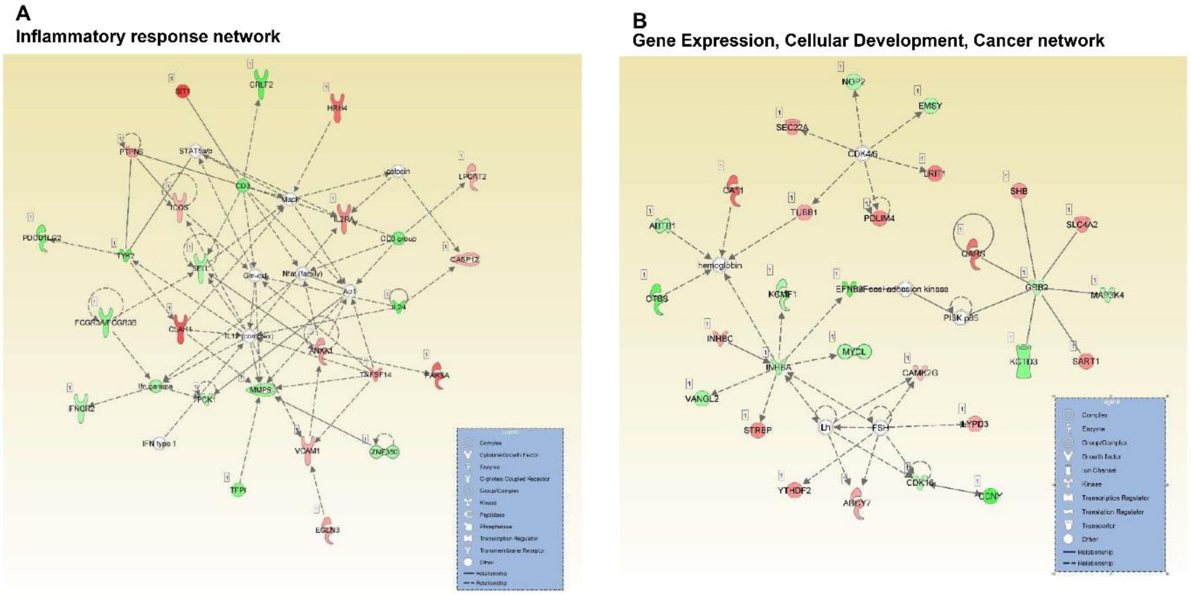

Figure S4. Network analyses of differentially-expressed genes in CD4⁺TIM-3⁺ and CD4⁺TIM-3⁻ TILs. Illustration of the inflammatory response (A) and gene expression, cellular development and cancer network (B) with predicted activated states of the network based on the transcriptomic data. Figure legend illustrates the relationship between molecules within the network.

Supplementary Table 1: **PCA loading analysis TIM-3^{+} vs TIM-3^{-}**

Gene Symbol	PC1	PC1(absolute)
PARP8	-0.02858	0.028581866
MAP4K3	0.028519	0.028519209
SEPT7P2	0.028496	0.028495642
RP1-130H16.16	0.028457	0.028457117
C7orf25	0.028397	0.028397325
ZNF587B	0.028348	0.028347773
NPHP1	-0.02833	0.028332759
UBTD2	0.028257	0.028257056
GABPB1	-0.02825	0.028254788
ZNF660	0.028238	0.028238114
EPHA10	0.028229	0.02822853
SEMA4F	0.028228	0.028228488
AQP12B	0.028187	0.028186531
CLEC4M	0.028153	0.028153019
FAM126B	0.02812	0.028120398
APCDD1	-0.02811	0.028110811
RP11-158I9.5	0.028108	0.028108387

EPB41L4A	-0.02811	0.028106025
PDE11A	0.028101	0.028100875
ANKRD34B	-0.02807	0.028066306
ELAVL2	-0.02805	0.028054469
ESRRG	-0.02805	0.028052295
RSRC2	0.028018	0.02801827
TMEM184A	0.028017	0.028017309
GTF2H5	0.028007	0.028007209
DSCR9	-0.02801	0.028006263
BCAT1	0.027991	0.027991028
C2orf49	0.027978	0.027977966
SMIM13	0.027975	0.027974839
RP11-3P17.4	-0.02796	0.027962199
DDX54	-0.02794	0.02794464
MEGF11	-0.02794	0.027938261
SLC35B4	-0.02791	0.027914405
TRIP4	-0.0279	0.027900571
PLAG1	0.027898	0.027898402
SERPING1	0.027872	0.027871846
MRVI1	0.027871	0.027871297
C11orf30	0.027867	0.027867262
RP11-567M16.1	-0.02783	0.027828973
SPATA6L	-0.02783	0.027826589
STK10	-0.02783	0.027826088
DAGLB	0.027823	0.027823037
PSMC2	-0.02777	0.02776921
ARHGAP42	0.027756	0.027756045
UNC5D	0.027731	0.0277311
KIAA0319L	-0.02767	0.027668191
OR4D1	-0.02765	0.02764819
IREB2	0.027629	0.02762868
NF1	0.027621	0.027621368
CNNM1	-0.02761	0.027612864
ZNF324B	-0.02761	0.02761156
PTAFR	0.027577	0.027576967
OR6K6	-0.02756	0.027561737
ARL17A	-0.02753	0.027527973
COQ5	-0.02752	0.027521689
FAM169B	-0.02751	0.027514951
ZNF185	0.027513	0.027513375
SPATA9	0.027488	0.02748824
ZBTB21	0.027485	0.027485263
CRTAP	-0.02748	0.027482871
CX3CR1	0.027451	0.027451033
S100A13	-0.02744	0.02743806
RGPD4	0.027435	0.027435211

PIGQP1	-0.02742	0.027422892
OS9	-0.02741	0.027411012
SUSD1	-0.02741	0.027406854
F11R	0.027403	0.027402995
C3orf58	-0.02738	0.027379407
C2	0.027379	0.027379403
ZNF891	-0.02737	0.027372455
ATAT1	0.027369	0.027368632
DDX41	-0.02736	0.027360723
ZNF560	-0.02735	0.027352873
TPTEP1	-0.02735	0.027350263
SLC35G1	0.027321	0.027320681
RP11-2O17.2	0.027317	0.027317272
CTD-2281E23.1	-0.02732	0.027316893
HSD17B6	-0.02731	0.027312228
PCCB	0.027307	0.027306938
HELLS	0.027302	0.027302167
GABRP	-0.0273	0.027300184
AP1G2	-0.0273	0.027295411
EXTL2	-0.02729	0.027291366
PWP2	-0.02728	0.027275085
SHPK_1	-0.02727	0.027270255
DENND6A	0.02726	0.027260164
KLHL9	0.027258	0.027258393
SLAIN2	0.02722	0.027220169
ANKS4B	0.027219	0.02721852
TCP11X2	-0.02722	0.027218011
RP11-351J23.1	0.027213	0.027213415
PTCD2	0.027208	0.027208155
RP11-536K7.3	0.027201	0.027201123
RP11-700P18.1	0.027199	0.027198876
SNHG17	-0.0272	0.027198254
SUPT20H	0.027194	0.027194307
VCL	0.027192	0.027191503
PPP4R1	0.027188	0.027187536
EZH2	0.027186	0.027185518
PBXIP1	0.027182	0.027181945
RPS13	-0.02718	0.027179585
RP11-448P19.1	0.027169	0.027168627
C12orf29	-0.02716	0.027162626
PABPC1P11	-0.02713	0.027129027
TMEM208	-0.02712	0.027122385
RP11-348M3.2	-0.02712	0.027121302
ZNF334	-0.02711	0.027107919
ZNF654	0.027108	0.027107752
SGMS1	0.027093	0.027093024

EMC3-AS1	-0.02709	0.027088847
PRKCB	0.027064	0.02706445
FAM219A	0.027048	0.02704804
PLXDC1	0.027037	0.02703659
MTMR14	-0.02703	0.027033522
ZNF418	-0.02703	0.027030714
XKR6	0.027015	0.027015206
EP400NL	0.027011	0.027010606
ZFP14	-0.027	0.027000154
KRT9	-0.027	0.026999424
CLRN3	-0.02698	0.026982671
AC093690.1	-0.02697	0.026970679
BEST1	-0.02694	0.026943876
BROX	-0.0269	0.026904168
INHBA	0.026899	0.026899463
TSPAN10	-0.0269	0.026896967
FAM161B	-0.02688	0.026883087
ZNF217	-0.02688	0.026882794
CEP76	-0.02688	0.026876537
ZNF460	0.026875	0.026875148
ZSCAN5A	-0.02687	0.026874146
CTB-134H23.3	0.026861	0.026860785
SCNN1G	-0.02685	0.02684691
RP11-96P7.1	-0.02685	0.026846052
RP5-1061H20.4	0.026839	0.026838501
OR52K1	0.026836	0.026836333
FKBP1B	-0.02683	0.026827415
ENTPD3	0.026812	0.026812367
CEP57	0.026806	0.026806117
UNC119B	0.026796	0.026796178
LAMA2	0.026791	0.026791314
RNF8	0.026783	0.026782542
HSPB3	-0.02678	0.026776707
WDR31	-0.02676	0.026763466
HOXD8	0.026751	0.026751007
MLK4	-0.02675	0.026750453
AC017002.2	0.026744	0.026743787
GPSM2	-0.02673	0.026728708
CCDC68	0.026724	0.026724043
DNAJB11	-0.02672	0.026720324
SRR	-0.02671	0.02670938
RBPJ	0.026697	0.026697301
PSG9	-0.0267	0.026696313
HRH4	-0.02669	0.026692184
OTUD7A	-0.02669	0.026691223
DGKI	-0.02667	0.026670913

C11orf83	0.02667	0.026670149
WBP2NL	0.026664	0.026663715
RAB3IL1	-0.02666	0.026655976
ETNK1	-0.02665	0.026651038
RP11-196E1.3	0.026641	0.026640843
SERP1	-0.02664	0.026639031
AKR1C2	0.026638	0.026638096
RP4-669H2.1	0.026637	0.026637442
ITGAV	0.026636	0.026635527
GPRC5C	0.026635	0.026634875
LINC00963	0.026633	0.026632679
RRP1	-0.02662	0.026623321
ZNF75A	-0.02662	0.026621394
UGT2B29P	-0.02662	0.026620992
AC008073.9	0.026607	0.026606537
AGXT2	-0.02661	0.026606483
MFSD12	0.026602	0.026601571
SPCS3	-0.02656	0.026560547
RPS17P14	0.026558	0.026558127
ZSCAN32	0.026537	0.026536756
RP4-756G23.5	-0.02653	0.026530518
NIPBL	-0.02651	0.026506352
POU2AF1	-0.0265	0.026504632
RP1-232N11.2	-0.0265	0.026496798
FSTL5	-0.02649	0.026486261
RPL7P6	-0.02647	0.026468289
LY6G5B	0.026461	0.026461164
SCN5A	-0.02646	0.026456946
BCAS1	0.026457	0.026456615
ARL8B	0.026455	0.026455025
MYO16	0.02645	0.0264501
POFUT1	0.026442	0.026441748
EXOSC9	0.026427	0.026427384
NUDT6	0.026423	0.026422658
MRPL17	0.026406	0.026406121
KLHL28	-0.0264	0.026399832
CCDC70	-0.02639	0.026394184
SSX2IP	-0.02639	0.02639147
RP11-131J3.1	0.026385	0.026385266
ETV5	0.026384	0.026383681
BEND4	0.026381	0.026380528
CTB-73N10.1	0.026354	0.026354408
NDEL1	0.026349	0.026349102
CRYBG3_1	0.026346	0.026346314
PPAPDC2	-0.02634	0.026339611
THAP9-AS1	0.026331	0.026331028

DMTN	-0.02632	0.026323124
PYROXD1	0.026321	0.026320945
SLC26A9	-0.02631	0.026313692
RP11-770J1.4	0.026313	0.026312588
HEBP2	0.026306	0.026305925
RP11-1197K16.2	0.026305	0.026305089
RPS16	-0.02629	0.026288424
CD48	-0.02628	0.026284139
RP5-826L7.1	0.026283	0.026282611
GTPBP2	-0.02628	0.0262802
CTBS	0.026277	0.026277473
PRPF4	-0.02627	0.026272262
MEST	-0.02626	0.026259838
TRIM64FP	0.026253	0.02625266
DHRS1	-0.02625	0.026247515
AC128709.1	0.026234	0.026233676
LINC00708	-0.02623	0.026231489
UBE3C	0.026228	0.026227913
KLRG1	-0.02622	0.026224641
RP1-310O13.7	-0.02622	0.026223456
ZCWPW1	0.02622	0.026220316
TANGO2	-0.02622	0.026215219
ZNF860	-0.02621	0.026206535
RP11-356C4.5	0.026185	0.026184912
BCL11A	0.026184	0.026184493
TCF20	-0.02618	0.026178359
EIF4ENIF1	-0.02617	0.026166024
TM9SF1_1	0.026143	0.02614319
AZGP1	0.026143	0.026142803
HPS3	0.026136	0.026135763
C15orf57	0.026134	0.026134198
GOSR2	0.026127	0.026126813
SRD5A2	0.02612	0.026119547
C1orf106	0.02611	0.026109725
RP11-51B23.3	-0.0261	0.026104072
DCX	-0.0261	0.026098287
MYNN	-0.0261	0.026098198
SOX30	0.026097	0.026096768
ZNF347	-0.0261	0.026095946
ANKRD50	-0.02609	0.026094255
DTD1	0.02608	0.026080482
SNHG1	-0.02608	0.026076102
RP13-516M14.4	-0.02607	0.026066206
TMEM178B	0.026052	0.026051956
AMICA1	0.026046	0.026046432
TEX36-AS1	0.026041	0.026040835

USP49	0.026031	0.02603071
RP13-39P12.3	0.026024	0.026024136
MT1JP	0.026018	0.026017842
PPP6R3	0.026008	0.026008352
XPC	0.026008	0.026007819
RP11-435B5.7	0.026005	0.026005151
C5AR1	-0.02601	0.0260051
RP13-631K18.3	0.025994	0.025993779
FAM129A	0.025986	0.025986138
CHST9	-0.02599	0.025985647
OSBPL1A	0.025978	0.025977564
REG1B	-0.02596	0.025964885
TNFSF14	-0.02596	0.025963497
TFAM	0.025962	0.025961599
RP11-218M22.1	0.025931	0.02593133
ELOVL4	0.025913	0.025913461
GLUD1	0.025906	0.025905938
AC006159.3	0.025899	0.025898612
PTPN4	-0.02589	0.025892769
RGL4	0.02588	0.02587964
ATCAY	-0.02588	0.025877348
GGTLC3	0.025876	0.025876033
RNF207	-0.02587	0.025868827
RARB	0.025868	0.025867904
PARP4	-0.02586	0.025863866
RP11-506O24.1	-0.02586	0.025858992
C4orf33	0.025858	0.025858496
KIAA1407	-0.02586	0.025855442
AKAP3	0.025852	0.02585232
MRPL20	-0.02584	0.025842456
TRIM24	-0.02584	0.025836386
ZNF22	-0.02583	0.025834938
CYP2R1	0.025834	0.02583428
AC010096.1	-0.02583	0.025833094
RP11-53B2.3	0.025823	0.025823466
PCOLCE2	0.025813	0.025813426
SEMA6C	-0.02579	0.025791905
LINC00469	0.025786	0.0257863
ZSCAN18	-0.02578	0.025779123
ZNF552	0.025772	0.025772429
RP11-214L13.1	0.025756	0.025755806
ACSF3	-0.02575	0.025746404
FBXO4	0.025722	0.025721926
TFPI	0.025718	0.025718184
VAC14-AS1	0.025713	0.025713385
NOL11	0.02571	0.025710368

RP11-110I1.12	-0.02571	0.025709887
CIDEB	0.025697	0.02569654
WHAMMP2	0.025693	0.025693457
SKP1	0.025679	0.025679375
CDK2	0.025677	0.025677491
FOSB	0.025675	0.025675324
STAU2-AS1	-0.02567	0.02567432
RTKN	0.02567	0.025669589
FAM221A	-0.02565	0.025652239
RAB3IP	-0.02564	0.025637094
C10orf85	-0.02564	0.025636824
IRF4	0.025632	0.025631989
C1orf35	0.025629	0.025629076
MINPP1	0.025628	0.025628392
TTC12	0.025624	0.02562396
PRCC	-0.02562	0.025623896
ENOX1-AS1	0.02562	0.025619548
ARL4A	0.025612	0.025612132
SLC27A2	0.025605	0.025604961
RP11-192P3.5	-0.0256	0.025600616
ACSF2	-0.0256	0.025596576
PBK	0.025587	0.025586548
RP11-417N10.3	0.025583	0.025583111
UNC45B	0.025577	0.025576796
HNRNPK	0.025575	0.025574994
MED15P9	0.025558	0.02555806
LETM2	0.025548	0.025547635
OSER1-AS1	0.025547	0.025546999
SLC5A9	-0.02553	0.025532866
KRTAP4-1	-0.02553	0.025532189
CCDC81	0.025532	0.025532098
WDR93	0.025529	0.025528974
DCTN3	-0.02553	0.025527885
SPDL1	0.025526	0.025525938
MCM5	-0.02552	0.025521394
SLC39A11	-0.02552	0.025519117
RP11-283G6.4	0.025514	0.025514154
INSIG1	-0.02551	0.025512878
CHRAC1	-0.02551	0.025511277
IDO1	0.02551	0.02551022
HIPK3	-0.0255	0.025499345
SMU1	0.025497	0.025496698
TECTB	-0.02549	0.025486165
WDR26	-0.02548	0.025482743
CD163L1	0.025479	0.025479262
NSMCE1	-0.02548	0.025478913

CDK2AP1	0.025478	0.025477882
DOK6	-0.02547	0.025473172
ELOVL6	0.025445	0.025444606
PRELID1	0.025444	0.025443536
ADAMTS1	-0.02544	0.025442914
SMAD9	0.025443	0.025442857
PSMD5	-0.02544	0.025441
MCM3	-0.02544	0.025436101
KLB	-0.02544	0.025435219
ZNF625	-0.02543	0.025433331
CTC-471F3.5	0.025421	0.025420716
RPL5P27	0.025416	0.025415978
SLC30A5	-0.02541	0.025409385
PHYH	0.025405	0.025405368
EHD2	-0.0254	0.025403059
TBPL2	-0.0254	0.025396793
ZNF429	-0.0254	0.025395272
CTC-360J11.4	-0.02539	0.025393728
STX3	0.025388	0.025387705
EIF3M	0.02537	0.025369586
RP5-1109J22.2	-0.02536	0.025364227
TEX261	0.025361	0.025361236
GAPVD1	0.02535	0.025349844
TBL1XR1	0.025349	0.025348792
RP11-308N19.4	-0.02535	0.02534567
CTD-3064H18.4	0.025316	0.025316462
RP11-175K6.1	-0.0253	0.025301928
C2orf16	0.025299	0.025299324
CIDEC	-0.0253	0.025297437
EBLN2	0.025289	0.025288558
WNT2B	-0.02528	0.025283263
NTN5	-0.02527	0.025269506
C12orf57	-0.02527	0.025267149
MCCC1	-0.02527	0.025266463
RAB36	-0.02527	0.025265893
RP11-55K22.5	0.025259	0.025259446
GOLGA6L4	0.025259	0.025258527
EDNRB	0.025257	0.025257053
HIST1H1D	0.025256	0.025255828
RP11-122M14.1	0.025248	0.025248307
MNDA	0.025245	0.025245484
ZNF550	-0.02524	0.025243949
LIMK2	0.025243	0.025242904
EIF2B2	0.025234	0.025233531
SNPH	-0.02523	0.025233505
SETD5	0.025228	0.025227813

PPP1R12B	0.025223	0.025222874
FBXO40	0.025209	0.025209009
PRODH	-0.02521	0.025207181
TBC1D25	-0.0252	0.025202087
ZNF835	-0.02519	0.025188512
OBSCN	-0.02518	0.025183496
ACTR8	0.02517	0.025170481
ASS1	-0.02517	0.025169916
CTC-507E2.1	-0.02516	0.025159209
RCHY1	0.025158	0.025157943
FGGY	-0.02516	0.025157298
CYP11B2	-0.02515	0.025152637
PCM1	0.025145	0.025145479
ZSWIM7	0.02514	0.025140031
AC141586.5	-0.02514	0.025137449
NGFR	0.025135	0.025135414
KCMF1	0.025135	0.025134907
RP11-382J12.1	0.025132	0.025132238
C12orf66	-0.02513	0.025128545
AMER3	0.025127	0.025127137
ZNF143	0.025126	0.025125774
AGAP11	-0.0251	0.025095352
CDK10	-0.02509	0.025093501
TRAM2-AS1	0.025089	0.025088502
GABPA	0.025085	0.025085345
NEMF	0.025084	0.02508386
CTB-131B5.5	0.025081	0.025081293
MYOM3	-0.02507	0.025074275
AL138847.1	0.025059	0.025058551
UTP14C	-0.02506	0.025055167
METTL21B	0.025051	0.025050617
CTA-217C2.1	-0.02505	0.025048134
ABCB4	-0.02504	0.025042791
QARS	-0.02504	0.025037361
GNAZ	-0.02504	0.025035597
CHRNA7	-0.02503	0.025027858
RIMS3	0.025015	0.025015341
USF2	0.025013	0.025012534
MOB1A	0.025007	0.025007112
LINC00704	0.025003	0.025002551
ZKSCAN7	-0.025	0.025001492
RP11-678G15.2	-0.025	0.02499725
CTD-2023N9.1	-0.02499	0.024988843
AQP4-AS1	0.024988	0.024987793
RNF13	0.024984	0.024984108
FBXO33	-0.02498	0.024981963

ARHGAP28	0.02498	0.024980232
C9	-0.02497	0.024971022
TMEM40	-0.02497	0.024969842
MGAT1	0.024968	0.024968244
KIF9	0.024966	0.024965598
WDR82	0.024957	0.024956508
VPS37C	-0.02495	0.024947394
MOGS	0.024945	0.024945207
RP4-811H24.9	0.024941	0.024940824
BCL6B	0.024939	0.024939187
BAALC	-0.02494	0.024936312
C1orf68	0.024935	0.024935413
AC000036.4	-0.02493	0.02493366
IL23R	0.024933	0.024933059
EEPD1	-0.02493	0.024925145
CTA-246H3.8	-0.02492	0.024923261
DAG1	0.024919	0.024918905
AC132216.1	0.024899	0.024898631
ELAVL4	-0.02488	0.02488221
SIK3	-0.02487	0.024870922
SLC2A9	0.024868	0.024867628
RP3-514A23.2	0.024865	0.024865421
CALCB	0.02486	0.024860349
MXD1	-0.02485	0.02485154
SLC1A3	-0.02485	0.024849549
SDR9C7	0.024849	0.024848772
FAM205CP	-0.02483	0.024834363
EIF1	-0.02483	0.024830823
PTPN13	0.024827	0.02482692
IQCH-AS1	-0.02483	0.024826015
RP5-884M6.1	0.024825	0.024825054
IGFBP4	0.024824	0.024824483
RP11-262H14.4	-0.02482	0.024823493
EPDR1	-0.02482	0.024817362
METTL13	0.024805	0.024804546
TUBA4A	-0.0248	0.02479791
TNFSF4	-0.02478	0.024783194
MDN1	-0.02477	0.024774985
TMEM168	-0.02476	0.02475623
RP3-473B4.3	0.024755	0.024754902
KIAA0907	0.024737	0.024736693
PANX2	-0.02474	0.024736528
THUMPD3	0.024736	0.024735877
GOLGA8G	-0.02473	0.024731766
ARMC1	-0.02472	0.024720548
CCDC63	-0.02472	0.024719466

CLVS1	0.024716	0.02471603
PLA2G2A	-0.02471	0.024714437
MDH1B	-0.02471	0.024712822
WDR37	0.024709	0.024709002
MRPS24	0.024706	0.024705936
RP11-106M3.3	0.024699	0.024699278
JKAMP	-0.02469	0.024688582
TATDN2	0.024682	0.024682251
ELFN2	-0.02468	0.02468139
OR51H1P	-0.02468	0.024680358
FAM225A	-0.02467	0.024674784
WDR72	-0.02467	0.024674609
CACUL1	0.024674	0.024673596
ARSD	-0.02466	0.024662519
LY6E	-0.02466	0.024658837
KDM3A	-0.02465	0.024651456
TMEM17	-0.02464	0.024635482
CSGALNACT2	-0.02463	0.024625188
ANAPC4	0.024622	0.024621926
NRG1-IT1	-0.02461	0.024605236
RP11-50C13.2	0.024601	0.024600908
ZNF559	0.024589	0.024588572
URB1	-0.02459	0.024587111
